# Molecular Research on Polycystic Ovary Syndrome (PCOS)

**DOI:** 10.3390/biomedicines11051358

**Published:** 2023-05-04

**Authors:** Simona Daniele, Elisa Chelucci, Giorgia Scarfò, Paolo Giovanni Artini

**Affiliations:** 1 Department of Pharmacy, University of Pisa, 56126 Pisa, Italy; simona.daniele@unipi.it (S.D.); e.chelucci@studenti.unipi.it (E.C.); 2 Division of General Medicine, Department of Clinical and Experimental Medicine, University of Pisa, 56126 Pisa, Italy; g.scarfo1@studenti.unipi.it; 3 Division of Gynecology and Obstetrics, Department of Clinical and Experimental Medicine, University of Pisa, 56126 Pisa, Italy

Polycystic ovary syndrome (PCOS) is an endocrine systemic disorder with a prevalence of between 5% and 20% that commonly affects childbearing-aged women [1,2,3]. Principally, PCOS is characterized by a hormonal and metabolic imbalance leading to the formation of water-retained cysts in ovarian follicles, chronic oligo/anovulation, and hyperandrogenism (biochemical/clinical signs) [3,4,5,6,7].

Due to syndrome heterogeneity, PCOS women generally exhibit not only gynecologic manifestations, but also different metabolic and endocrine impairments, including insulin resistance (IR). Furthermore, PCOS often involves more morbidities such as psychiatric disorders, obstructive sleep apnea, disordered eating, sexual and thyroid dysfunctions, acne, alopecia, and hirsutism, impairing quality of life.

This broad and varied set of clinical dysregulations needs to be carefully examined because they commonly lead to an increased risk of cardiovascular diseases (CVDs), type 2 diabetes mellitus (T2DM), metabolic syndrome (MetS), nonalcoholic fatty liver disease/nonalcoholic steatohepatitis (NAFLD), atherosclerosis, obesity, autoimmune diseases, infertility, endometrial carcinoma, and adverse pregnancy outcomes (miscarriage, preterm birth, pre-eclampsia, or gestational diabetes).

PCOS is a complex polygenic and multifactorial syndrome, whose development can be influenced by individual predispositions, precise clinical sub-phenotyping, age, BMI, epigenetic, and environmental factors. However, PCOS etiopathogenesis still remains unclear; the relative signaling pathways and molecular mechanisms need to be largely investigated, focusing on possible interactions between genetic and environmental factors.

The Special Issue described herein offers a wide overview of the current knowledge on the progression of PCOS from present clinical evidence to future perspectives across a whole series of research articles, papers, and reviews primarily focused on the evaluation of the involved molecular mechanisms and signaling pathways and aimed to find innovative counteracting therapies.

Among PCOS’s remarkable manifestations, endocrine imbalance and related risk factors have been extensively researched via clinical, in vivo, or in vitro studies.

In the systematic review by Glintborg et al. [2], evidence concerning TD2M development risk in normal-weight PCOS women was discussed, with a focus on insulin resistance, β-cell dysfunction and the possible moderating effect of PCOS phenotypes (hyperandrogenism, age, and ethnicity). The authors provided recommendations regarding prospective screening for TD2M in these women by considering individual risk markers such as hyperandrogenism, age > 40 years, Asian ethnicity, and weight gain.

Furthermore, Glintborg et al. [4] conducted a national cohort study with the aim of studying diabetes as a mediator for depression in PCOS. Indeed, this register-based study documented a 40% increase in depression risk in Danish PCOS women toward age-matched healthy controls, of which 12.5% could be related to diabetes. Moreover, these findings highlight the tendency of PCOS women to develop depression, exacerbated by overweight, infertility, hyperandrogenic phenotypes, medical comorbidities, or low family income. A predisposition to autoimmune thyroiditis is also very common. In this regard, Feigl et al. [8] performed a cross-sectional observational study including pregnant women without PCOS to evaluate the comparability of mothers and offspring thyroid parameters, focusing on their impact on pregnancy and neonatal outcomes. Perinatal complications and thyroid dysfunctions were more frequent in the PCOS cohort, but no significant differences in thyroid parameters between women or neonates without complications were reported. Nonetheless, PCOS women showed free triiodothyronine (fT3) levels significantly lower than controls, and higher thyreoperoxidase antibody (TPO-AB) levels were observed in PCOS women and their neonates, leading to a higher prevalence of hypothyroidism in these women. This study shows the importance of thyroid evaluation and monitoring throughout pregnancy, mainly in PCOS women.

Related to the PCOS endocrine imbalance are also gonadotropin dysfunctions (high serum AMH and LH, normal or low FSH levels with increased LH/FSH ratio > 2.5), all of which potentially compete with pathogenesis. Therefore, Gargus et al. [9] mimicked typical PCOS gonadotropin stimulation in vitro to evaluate subsequent changes in ovarian steroidogenesis. They exploited a single-tissue model consisting of murine ovarian explants, overcoming all uncontrolled variables due to syndrome heterogeneity in vivo. This enabled the iterative, systemic study of mechanisms triggered by various etiologic factors, which can contribute to PCOS phenotypes. Moreover, they developed a high-throughput LC–MS/MS technique to study steroid metabolome alterations due to the three selected gonadotropin milieus, measuring seven ovarian steroids released in the conditioned culture media. This first-of-its-kind exhibition of hormone levels from single tissues could provide promising prospects for evaluating other potential insults in PCOS pathogenesis within the same experimental framework.

On the other hand, Abdul Hamid et al. [5] explore the current knowledge on sex steroid receptors (ER, PR, and AR), their pathophysiological role in PCOS phenotypes, and their consequent use as valid therapeutic targets. Even the overexpression of the anti-Müllerian hormone (AMH) contributes to PCOS pathogenesis, increasing levels of androgens, the follicles cohort, and the promotion of anovulation. Aside from this evidence, AMH overexpression also found in the male PCOS equivalent, and its possible effects on the reproductive function of male relatives of PCOS women were discussed by Di Clemente et al. [10]. Reproductive and metabolic disorders in PCOS women could be caused by alterations during fetal and pre-pubertal life. A recent hypothesis suggested that high AMH levels during PCOS pregnancy, and consequent fetus androgenization, could lead to PCOS in adulthood. Indeed, first-degree male relatives could present with a male PCOS equivalent, and metabolic disturbances are the main traits. In this regard, Siemienowicz et al. [11] examined hepatic mitochondrial function and lipid metabolism in adolescent prenatally androgenized (PA) males from a PCOS ovine model, to whom testosterone was administrated in order to generate a prenatal androgenic overexposure. Indeed, previous studies supported the association between the latter and PCOS phenotype development in adult life. Thus, the authors aimed to correlate the prenatal excessed androgen exposure to NAFLD development (independent of diet or obesity) in first-degree male relatives of PCOS sufferers who exhibit a PCOS-like metabolic phenotype. This study found an increased accumulation of hepatic cholesterol and fatty acids (FA), dual glucose and FA metabolism impairments, altered mitochondrial transport and mitophagy, reduced oxidative phosphorylation and ATP synthesis in adolescent PA. Cholesterol or FA overload contributes to mitochondrial dysfunction. This is associated with oxidative stress and increased hepatic ROS, which could lead to NAFLD progression. These preliminary findings indicate that males born from hyperandrogenic pregnancies could develop an increased risk of hepatic diseases.

Regarding androgenic dysfunctions, a concept paper by Gleicher et al. [12] reclassified the current PCOS phenotypes (A, B, C, and D under the Rotterdam criteria) into two different phenotypes: a hyperandrogenic phenotype (H-PCOS) (characterized by metabolic abnormalities) a hyper-/hypoandrogenic phenotype (HH-PCOS) (distinguished by advancing age and a hyperactive immune system mostly caused by autoimmunity and inflammation).

PCOS metabolic anomalies are demonstrated to be related to incorrect element metabolism. A clinical study by Szczuko et al. [13] examined the degree of dependence between erythrocytes potassium concentration, saturated fatty acids (SFAs) levels, and inflammatory mediators in PCOS women. Although the involved processes warrant further investigations, the authors explain that increased SFAs in red-blood-cell membranes reduced potassium influx by destabilizing the cytosol pH and increasing the inflammatory response through the activation of the AA (arachidonic acid) cascade, in which the 9, 13 HODE (derivatives synthesized from linolenic acid) and 5LOX pathways are mainly involved. Furthermore, epidemiological studies linked low serum potassium to a significant CVDs risk. Concerning this, Nawrocka-Rutkowska et al. [14] confirmed through a clinical study the positive correlation between oxidative stress markers and the occurrence of CV complications in PCOS women. They proposed AIP and Castelli Index as atherogenicity additional indicators to assess the risk of cardiometabolic diseases in PCOS in order to rapidly identify risk groups among this population. Niepsuj et al. [15] took into account further parameters, such as oxidized low-density lipoproteins (oxLDL-C) and the ferric-reducing ability of plasma (FRAP), to evaluate pro/anti antioxidant imbalance blood in PCOS patients. This clinical study found a significant association between oxLDL concentration and the FRAP value, and selected hormonal and metabolic parameters in the PCOS course. Indeed, the latter one together with the syndrome development is deeply influenced by increased oxidative stress. Additionally, it has been reported that ROS are involved in genetic changes as well as PCOS development.

Although little is known about molecular mechanisms and signaling pathways involved in PCOS pathogenesis [3], several functional studies on genetic variance and epigenetic changes have broaden our knowledge on these topics. In this regard, due to the different proteins and molecular pathways involved, a genetic approach could be a non-pharmacological method for improving early PCOS diagnosis, prognosis, and treatment, as reviewed by Nautijal et al. [6]. The latest investigations reported PCOS as a multigene-origin disease. PCOS gene mapping enables the identification of susceptible loci, thus leading to the discovery of novel disease pathways and drug targets. Moreover, the individuation of key gene polymorphism is useful in the timely diagnosis and screening of PCOS subtypes.

Genome-wide association studies (GWAS) have found several genetic loci associated with PCOS and its clinical features. Lidaka et al. [16] investigated the prevalence of five single-nucleotide variants (SNVs) in the *YAP1* gene among PCOS adolescents compared to healthy cases, evaluating any possible correlation with PCOS clinical manifestations. Indeed, it has been observed that the *YAP1* gene plays a key role in PCOS pathogenesis and relative metabolic diseases.

Alternatively, Scarfò et al. [3] reviewed the literature on molecular and epigenetic mechanisms involved in PCOS, focusing on the positive role of diet and physical activity as non-pharmacological interventions in the syndrome management. Their study does not focus on a single topic but provides a wide overview of PCOS pathogenesis based on epigenetic mechanisms, outlining beneficial effects of diet and physical activity on counteracting the clinical manifestations of disease. The importance of next-generation genetic analysis was underlined by the authors to improve nonpharmacological strategies; likewise, necessary lifestyle changes were encouraged. Related to epigenetics in PCOS, in their systematic review, Vitale et al. [17] discussed miRNA as potential PCOS diagnostic markers and therapeutic targets. Some miRNAs have been found in serum, follicular fluid, theca, and granulosa cells, regulating glucose and lipid metabolism, hormone synthesis, and follicular maturation. In this sense, an altered miRNA profile could partially explain PCOS heterogeneity. Among the therapeutic approaches discussed, this Special Issue provides another two reviews, which outline how weight gain and IR contribute to exacerbating syndrome-related disorders, providing new perspectives in treatments for PCOS patients. Petrillo et al. [7] discuss a wide range of putative compounds as complementary substances able to overcome IR. Similarly, Jensterle et al. [18] debate the therapeutic potential of glucagon-like peptide-1 agonists (GLP-1Ras) in promoting weight loss in PCOS, for which a fatty excess represents an important factor risk. Despite this research, clinicians still do not completely recognize GLP-1Ras use in PCOS; a weight-centric approach using GLP-1Ras could support the existing options for its management.

In summary, this Special Issue tries to understand PCOS pathophysiology via the study of molecular and epigenetic mechanisms, individuate potential diagnostic markers and therapeutic targets, improve syndrome management, and encourage non-pharmacological strategies.
